# Assessment of several hydrodynamic properties of sugar beet (*Beta vulgaris* L.)

**DOI:** 10.1002/fsn3.1856

**Published:** 2020-09-07

**Authors:** Bijan Khorshidpour, Masoud Honarvar, Hossein Ahmadi Chenarbon

**Affiliations:** ^1^ Department of Food Science and Technology, Science and Research Branch Islamic Azad University Tehran Iran; ^2^ Department of Food Science and Technology, Science and Research Branch Islamic Azad University Tehran Iran; ^3^ Department of Agronomy College of Agriculture, Varamin ‐ Pishva Branch, Islamic Azad University Varamin Iran

**Keywords:** *Beta vulgaris* L, falling time, hydrodynamic properties, physical properties, sugar beet, terminal velocity

## Abstract

It is necessary to know the hydrodynamic properties of agricultural products in order to analyze the behavior of materials when transporting and grading. In this study, the samples were divided into three groups based on their mass. Based on the results, the mean length, mass, volume, density, average projected area, and shape coefficient were 21.5 cm, 408 g, 386 cm^3^, 1.05 g/cm^3^, 620.16 cm^2^, and 11.68 in samples with m < 500 g, 24.1 cm, 681.8 g, 627.95 cm^3^, 1.08 g/cm^3^, 876.29 cm^2^, and 11.95 in samples with 500 < m <1,000 g and 28.4 cm, 1,389.36 g, 1,240.5 cm^3^, 1.12 g/cm^3^, 1,402.73, and 12.15 in samples with m > 1,000 g. Moreover, the terminal velocity, falling time, buoyancy, and drag force were 0.12 m/s, 14.26 s, 3.79 N, and 0.2 N in the samples with m < 500 g, 0.16 m/s, 10.92 s, 6.30 N, and 0.29 N in the samples with 500 < m <1,000 g and 0.22 m/s, 7.94 s, 12.03 N, and 1.49 N in the samples with m > 1,000 g. It is noteworthy that the water–sugar beet density difference and the mass had the greatest effect on terminal velocity and falling time whereas shape coefficient did not significantly influence these properties.

## Graphical abstract

Hydrodynamic properties of agricultural products can be used as a basis in order to designing of suitable machines for sorting, cleaning, handling and storage. So in this research, some hydrodynamic characters of sugar beet such as terminal velocity, falling time, buoyancy force and drag force were investigated to manage in reduce waste at the postharvest stage.

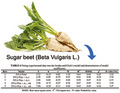

## 1 INTRODUCTION

Sugar beet (*Beta vulgaris* L.) is the most cultivated crop after sugarcane (*Saccharum officinarum* L.) for the production of sucrose for human consumption (Pan, Lu, Zhu, Tu, & Cen, 2016). Beetroot ingredients are divided into two main parts: about 75% water and about 25% solids. Solids can be divided into two parts: sucrose and nonsugar substances, part of which is water‐soluble and other water‐insoluble part, which is called the Mark. The Mark actually contains insoluble nonsugar materials in the cell wall, and most of it contains materials such as cellulose (21%–24%), hemicellulose (22%–31%), pectic substances (20%–40%), and lignin (2%–7%). On average, Mark make up about 4 to 5% of the beetroot's composition. Cellulose and hemicellulose give cell strength while pectic bodies, as cement, bind cells together (Lu, 2004). The extract is composed of sucrose and non‐sucrose‐soluble substances. Non‐sucrose‐soluble substances in sugar beet make up about 2.5% of the compounds. The most important non‐sucrose‐soluble substances include non‐nitrogenous organic matter, nitrogenous organic matter, and minerals. The waste during the harvesting, loading, and transporting of sugar beet is a major part of the quantitative and qualitative evaluation of this product. Direct and indirect impact and damage to the root of the sugar beet eventually cause the amount of sucrose stored in the root to be reduced and the residual amount not fully extracted (Pan, Lu, Zhu, McGrath, & Tu, 2015). The increasing food wastes are a serious challenge in most countries, and it is especially a challenge for developing countries, necessitating toughen national policies worldwide regarding food security and avoiding waste. In fact, wastes are a part of the agricultural products that could potentially be a part of household food portfolio but have failed to do so due to various reasons. A solution for maintaining the quality of the end product, reducing wastes, and bringing back the costs to national economies is to develop the processing industries and designing suitable equipment which are required for handling and cleaning. In this regard, it is necessary to understand the physical, chemical, hydrodynamic, and mechanical properties of agricultural products for designing of machines and equipment in order to obtain high‐quality products (Jahanbakhshi, Abbaspour‐Gilandeh, Ghamari, & Heidarbeigi, 2019; Rozbahani, Movahhed, & Ahmadi Chenarbon, 2019). At present, a huge number of devices for transporting, washing, and handling of different fruits are designed. Their functions are based on different characteristics of products such as color, dimension, weight, and density. In the same vein, a large number of studies were conducted on the effect of density as a good factor for grading fruits (Naderi–Boldaji, Fattahi, Ghasemi‐Varnamkhasti, Tabatabaeefar, & Jannatizadeh, 2008). At beginning, the density of fruit is calculated and then it is placed in the fluid flow. Then depending on the density of fruit which is either lower or higher than fluid's density, it can be floated on water or fall down. By using the separators and lifts which are placed properly, they can be graded. Although this is the most common method but it may cause problems because of using salts or other soluble materials in order to adjust the density of the water. In second common method, a vibrator is used and particles are separated based on the vibration without using water. However, because of its low sensitivity, it is not used very commonly. In third method, terminal velocity of fruits is used (Rozbahani et al., 2019; Topuz, Topakci, Canakci, Akinci, & Ozdemir, 2005). Terminal velocity is a constant speed of an object traveling in a fluid. Terminal velocity means the highest velocity that an object can reach when moving downward in a fluid. It happens when the sum of the drag and buoyancy forces is equal to the downward force caused by the gravity. Among hydrodynamic characteristics, the importance of terminal velocity for agricultural products is twofold. First, it is used to determine the velocity necessary to keep the object suspended, in a fluid which transfers it. Second, it shows the necessary velocity of fluid to separate objects.

So far, several studies have been carried out on determining the hydrodynamic properties of different agricultural products (Hasanpour Kahnamuyi & Ghaffari, 2013; Kheiralipour et al., 2009; Naderi–Boldaji et al., 2008; Rozbahani et al., 2019; Taheri Garavand, Rafiee, Keyhani, & Mirzaee, 2010). It is noteworthy that the density affects the terminal velocity. Fruits with different velocities reach different depths or heights in a liquid at a given time. So, by using a separator which is situated properly, fruits can be sorted (Mirzaee, Rafiee, Keyhani, Emam‐Djomeh, & Kheiralipour, 2009; Hasanpour Kahnamuyi et al., 2013; Moradi, Mousavi Khaneghah, Parvaresh, & Balanian, 2019; Rozbahani et al., 2019). Jordan and Clark (2004) mentioned using terminal velocity as a method for sorting fruits when the fruits are moving in a fluid with a density not equal to target density. Fruits with different terminal velocities reach different depths after moving down for a fixed distance in a fluid. In this situation, they can be then picked by dividers which are placed properly. In this approach, water can be a sorting medium and has lots of advantageous like low corrosion, easy disposal difficulties, and no need for adjusting its density. On top of that there is no need to change the fluid density and one can adjust the divider position for separation threshold setting purely mechanically (Jordan & Clark, 2004). Increasing demand for food has led to the demise of trade borders and the high volume of food commodities in the world. Effective presence on the world market and competing with other countries in exporting products require further development and advancement in postharvest technologies. In this regard, quality assessment and evaluation of technological characteristics of agricultural crops, as one of the important postharvest activities, has received more attention. According to the content, in this research, the use of sugar beet terminal velocity as a parameter for density‐based hydraulic grading to be used in the design processes of grading systems.

Simultaneously, the terminal velocity and falling time regression equations of sample movement were fitted according to their physical properties for finding the best model. No research, to the best of our knowledge, has been conducted on modeling of hydrodynamic properties of sugar beet, which was examined in the present study.

## 2 MATERIALS AND METHODS

Sugar beet samples were obtained from experimental field of Qazvin sugar factory at Qazvin province (Iran), during the 2019 harvest season. The beetroot samples, 250 in total, were selected based on Cochran's sampling method and after washing were stored in refrigerated storage at 4°C and 80% relative humidity prior to the experiment. The samples were without physical damage and free of physiological decay. In the following, physical and then hydrodynamic properties of each beetroot were measured at temperature of ∼25°C and 49% relative humidity in the laboratory of food physical properties of Science and Research Branch, Islamic Azad University.

### 2.1 Physical properties of beets

#### 2.1.1 Mass

The beetroot mass was measured using a digital balance with 0.001 g accuracy (Sartorius, model PT210, Germany).

#### 2.1.2 Volume

In order to calculate the beet volumes, water displacement method was used (Equation 1) (Mohsenin, 1986). The main advantage of the water displacement method is that it allows finding the volume of irregular solids. Also, this method is quick and easy if the correct liquid is used with the right object. But disadvantages of this method are that the object being immersed in the water may react with water or even dissolve slightly therefore reducing the accuracy of the results and may be too light and float on the water.

(1) $$v=\frac{m_{w}}{\rho_{w}}$$

where $m_{w}‐\;\;‐$displacement water mass (kg), $\rho_{w}‐\;\;‐$water density (kg/m^3^), and *v* ‐‐volume (m^3^).

#### 2.1.3 Dimensions

The beet dimensions a (length) and b (width) were determined using a digital caliper (Mitutoyo Co., Japan, ±0.01 mm).

#### 2.1.4 Density

The density of beetroots is obtained by Equation 2 (Taheri Garavand et al., 2010).

(2) $$\rho_{f}=\frac{m}{v}$$

where $\rho_{f}‐\;\;‐$beets true density (kg/m^3^), *m* ‐‐mass (kg), and *v* ‐‐volume (m^3^).

#### 2.1.5 Shape coefficient

The beets shape coefficient was measured from Equation 3 (Jalali, Ghaffari, Lotfi, & Akhunipour, 2013).

(3) $$S_{h}=\frac{A_{c}}{v^{\text{2}\;/\text{3}}}$$

where $S_{h}‐\;\;‐$shape coefficient (dimensionless), $A_{C}‐\;\;‐$average projected area was measured by digital image processing with 0.05 mm^2^ accuracy (m^2^), and *v* ‐‐volume (m^3^).

### 2.2 Hydrodynamic properties of beets

To determine some hydrodynamic properties sugar beet such as terminal velocity and falling time, a glued Plexiglas column was constructed, with height‐‐1,800 mm and cross‐section‐‐600 × 600 mm. Based on standards, this column is optimum sized according to beet diameter which is approximately 20 percent of column diameter (Mirzaee et al., 2009; Rozbahani et al., 2019). The column was filled with water up to the 1,700 mm. In the following, beet samples were placed at the top of column and were released in the water. A Samsung (ST69) digital camera (30 fps) recorded the sample displacement from releasing point to the bottom of the water column, simultaneously. A video‐to‐image converter was used to record the whole beet movement footage‐‐from the beginning at the top of the column to the end at its bottom‐‐as images in separate files.

#### 2.2.1 Drag, buoyancy force, and drag coefficient

Buoyancy force and drag force were calculated using Equations 4 and 5, respectively. Accordingly, by using Stokes’ law (N_Re_ >) the drag coefficient can be determined from Equation (6). For 10^3^ < N_Re_ <2 × 10^5^, the drag coefficient is equal to 0.44 (Mohsenin, 1986; Singh & Reddy, 2006).

(4) $$F_{d}=0.5{\left(V_{t}\right)}^{2}\rho_{w}C_{D}A_{p}$$

(5) $$F_{b}=\rho_{w}vg$$

(6) $$C_{D}=\frac{K}{{\left(N_{\text{Re}}\right)}^{n}}$$

where *V_t_* ‐‐terminal velocity (m/s), v‐‐beet volume (m^3^), *g* ‐‐gravitational acceleration (m/s^2^), $C_{D}‐\;\;‐$drag coefficient, $\rho_{w}‐\;\;‐$water density (kg/m^3^), *F_b_* ‐‐buoyancy force (N), *F_d_* ‐‐drag force (N), *N*_Re_ ‐‐Reynolds’ number (dimensionless), $A_{p}‐\;\;‐$surface area of beet (m^2^), and K and n are constant factors of model.

#### 2.2.2 Reynolds number

Based on the Equation (7), the Reynolds’ number was measured (Altuntas & Yildiz, 2007).

(7) $$N_{\text{Re}}=\frac{\rho_{w}Vd}{\mu }$$

where *d* ‐‐beet diameter (m), *V* ‐‐beet velocity (m/s), N_Re_ ‐‐Reynolds’ number (dimensionless), *µ* ‐‐static viscosity of water (N.s/m^2^), and $\rho_{w}‐\;\;‐$water density (kg/m^3^).

#### 2.2.3 Terminal velocity

##### Terminal velocity of beet measured by water column

According to Equation (8), the terminal velocity of the beet samples was measured (Kheiralipour et al., 2009).

(8) $$V_{t}=\frac{70\times 10^{‐2}}{\left(0.04\times N\right)}$$

Where $V_{t}‐\;\;‐$terminal velocity (m/s) and *N* ‐‐number of pictures in vertical distance.

##### Terminal velocity of beet measured by KHAT4 model

The model of KHAT4 (model 9) was employed for determination of terminal velocity of nonspherical objects while down falling and N_Re_ > 1. It is worth to note that the difference between water and beet densities, beet volume, and its shape coefficient are the main factors that affect terminal velocity of samples in water (Kheiralipour et al., 2009).

From Equations (6) and (7), Equation (9) will result.

(9) $$C_{D}=\frac{K\mu^{n}}{V^{n}d^{n}\rho_{w}^{n}}$$

where *d* ‐‐beet diameter (m), $V‐\;\;‐\text{beet velocity}\;\left(m/s\right),$ $\rho_{w}‐\;\;‐$water density (kg/m^3^), $C_{D}‐\;\;‐$drag coefficient, *µ* ‐‐static viscosity of water (N.s/m^2^), and K and n are constant factors of model.

(10) $$ma=F_{w}‐F_{d}‐F_{b}$$

where $F_{w}‐\;\;‐$gravitational force (N), $F_{d}‐\;\;‐$drag force (N), $F_{b}‐\;\;‐$buoyancy force (N), m‐‐mass (kg), and a‐‐acceleration (m/s^2^).

(11) $$ma=mg‐0.5{\left(V_{t}\right)}^{2}\rho_{w}C_{D}A_{p}‐\rho_{w}vg$$

where m‐‐ mass (kg), a‐‐acceleration (m/s^2^), *V_t_* ‐‐terminal velocity (m/s), v‐‐beet volume (m^3^), *g* ‐‐gravitational acceleration (m/s^2^), $C_{D}‐\;\;‐$drag coefficient, $\rho_{w}‐\;\;‐$water density (kg/m^3^), and $A_{p}‐\;\;‐$surface area of beet (m^2^).

On the other hand, dividing Equation (10) by ($v\rho_{f})$, gives:

(12) $$a=g\left(1‐\frac{\rho_{w}}{\rho_{f}}\right)‐0.5\rho_{w}\;V_{t}^{2}\;C_{D}\;A_{p}/\left(v\rho_{f}\right)$$

where a‐‐acceleration (m/s^2^), g‐‐gravitational acceleration (m/s^2^), $\rho_{w}‐\;\;‐$water density (kg/m^3^), *V*_t_ ‐‐terminal velocity (m/s), $C_{D}‐\;\;‐$drag coefficient, $A_{p}‐\;\;‐$surface area of beet (m^2^), v‐‐beet volume (m^3^), and $\rho_{f}‐\;\;‐$beets true density (kg/m^3^).

Based on previous study, $\frac{A}{v}$can be computed directly as a function of diameter. By separating $\frac{A}{v}$into two parts: dimensionless shape factor ($S_{h}$) and size, the following relationship was obtained (Jordan & Clark, 2004).

(13) $$\frac{A_{p}}{v}=\frac{S_{h}}{\text{size}}=\left[\frac{A_{p}}{v^{\left(\frac{2}{3}\right)}}\right]/\left[v^{\frac{1}{3}}\right]$$

where $S_{h}‐\;\;‐$shape coefficient (dimensionless), v‐‐beet volume (m^3^), and $A_{p}‐\;\;‐$surface area of beet (m^2^).

And by knowing that diameter is equal to Equation 14:

(14) $$d=e{\left(\frac{6v}{\pi }\right)}^{\frac{1}{3}}$$

where *d* ‐‐beet diameter (m), v‐‐beet volume (m^3^), and e‐‐constant factor.

(15) $$a=g\left(1‐\frac{\rho_{w}}{\rho_{f}}\right)‐k\left(\frac{\mu^{n}S_{h}\rho_{w}^{\left(1‐n\right)}V_{t}^{\left(2‐n\right)}}{v^{\left(\frac{n+1}{3}\right)}\rho_{f}}\right)$$

where a‐‐acceleration (m/s^2^), *g* ‐‐gravitational acceleration (m/s^2^), $\rho_{w}‐\;\;‐$water density (kg/m^3^), *V_t_* ‐‐terminal velocity (m/s),v‐‐beet volume (m^3^), $\rho_{f}‐\;\;‐$beets true density (kg/m^3^), *µ* ‐‐static viscosity of water (N.s/m^2^), $S_{h}‐\;\;‐$shape coefficient (dimensionless), and K and n are constant factors of model.

Then, setting acceleration to zero in Equation (15), the terminal velocity of the samples becomes:

(16) $$V_{t}=k\frac{{\left(\rho_{f}‐\rho_{w}\right)}^{\left(\frac{1}{2‐n}\right)}v^{\left(\frac{n+1}{3\left(2‐n\right)}\right)}}{\mu^{\left(\frac{n}{2‐n}\right)}\rho_{w}^{\left(\frac{1‐n}{2‐n}\right)}S_{h}^{\left(\frac{1}{2‐n}\right)}}$$

where $\rho_{w}‐\;\;‐$water density (kg/m^3^), *V_t_* ‐‐terminal velocity (m/s),v‐‐beet volume (m^3^), $\rho_{f}‐\;\;‐$beets true density (kg/m^3^), µ‐‐static viscosity of water (N.s/m^2^), $S_{h}‐\;\;‐$shape coefficient (dimensionless), and *K* and *n* are constant factors of model.

(17) $$V_{t}=A{\left(\rho_{f‐}\rho_{w}\right)}^{b}v^{c}S_{h}^{‐d}+E$$

where $V_{t}‐\;\;‐$terminal velocity (m/s),v‐‐beet volume (m^3^), $\rho_{f}‐\;\;‐$beets true density (kg/m^3^), $\rho_{w}‐\;\;‐$water density (kg/m^3^), $S_{h}‐\;\;‐$shape coefficient (dimensionless), and A, b, c, d, and E‐‐constants of model.

##### Terminal velocity of beet measured by Jordan and Clark's model

In this model, beet mass and water–beet density difference were considered as the effective parameters in beet terminal velocity (model 18) (Jordan & Clark, 2004).

(18) $$V_{t}={\text{Am}}^{b}{\left(\rho_{f}‐\rho_{w}\right)}^{c}+D$$

where $V_{t}‐\;\;‐$terminal velocity (m/s), $\rho_{f}‐\;\;‐$beets true density (kg/m^3^), $\rho_{w}‐\;\;‐$water density (kg/m^3^), *m* ‐‐mass of samples (kg), and A, b, c, and *D* ‐‐constants of model.

#### 2.2.4 Falling time

##### Falling time of beet measured by water column

Digital camera could record 30 frames per second, and the duration between each still was 0.033s. By multiplying the number of images in 0.033 s for each sample, the falling time was determined.

##### Falling time of sugar beet measured by KHAT4 model

The travel speed of an object is the distance divided by the travel time (Equation 19).

(19) $$V_{t}=\frac{X}{T_{d}}$$

where $V_{t}‐\;\;‐$terminal velocity (m/s), X‐‐distance (depth) (m), and $T_{d}‐\;\;‐$falling time (s).

To model the falling time of the beet samples, Eq. 21 was used, which is a combination of Equations 16 and 19 (Kheiralipour et al., 2009).

(20) $$T_{d}=\text{KX}\left[S_{h}^{\left(\frac{1}{2‐n}\right)}{\left(\rho_{f}‐\rho_{w}\right)}^{\left(\frac{‐1}{2‐n}\right)}v^{\left(\frac{‐n‐1}{3\left(2‐n\right)}\right)}\right]$$

where $T_{d}‐\;\;‐$falling time (s), $\rho_{w}$—water density (kg/m^3^), X‐‐distance (depth) (m),v—beet volume (m^3^), $\rho_{f}$—beets true density (kg/m^3^), $S_{h}$—shape coefficient (dimensionless), and K and n are constant factors of model.

(21) $$T_{d}=A{\left(\rho_{f}‐\rho_{w}\right)}^{‐b}v^{‐c}S_{h}^{d}+E$$

where $T_{d}$—falling time (s), v—beet volume (m^3^), $\rho_{f}$—beets true density (kg/m^3^), $\rho_{w}$—water density (kg/m^3^), $S_{h}$—shape coefficient (dimensionless), and A, b, c, d, and E‐‐constants of model.

##### Falling time of beet measured by Jordan and Clark's model

According to Jordan and Clark's model (model 22), the falling time of beet was measured (Jordan & Clark, 2004).

(22) $$T_{d}={\text{Am}}^{b}{\left(\rho_{f}‐\rho_{w}\right)}^{c}+D$$

where $\rho_{f}$—beets true density (kg/m^3^), $\rho_{w}$—water density (kg/m^3^), m—mass of beet (kg), and A, b, c, and D—constants of model, and $T_{d}$—falling time (*s*).

### 2.3 Statistical regression models

The nonlinear regression method was used in MATLAB for fitting data and in order to determine the reliability of the fits, in addition to determining R^2^, RMSE (root mean squares error), $X^{2}$ (chi‐square test), and P‐value (probability value) were used. The RMSE, root mean square error, gives the deviation between the predicted and experimental values.

## 3 RESULTS AND DISCUSSION

### 3.1 Physical properties of sugar beet

Tables 1 and 2 show some of physical and mean comparison results of data from hydrodynamic properties analysis of sugar beet samples at three different mass levels.

**Table 1:** Some of physical characteristics of sugar beet samples in three mass levels

Parameters	m < 500(g)			500 < m<1,000(g)			m >1,000 (g)		
	Min	Max	Mean	Min	Max	Mean	Min	Max	Mean
Length (mm)	149	243	215.142	226	256	241.09	210	336	284.500
Width (mm)	81	91	85.285	91	119	104.09	106	145	121.000
Mass (g)	322	493	408.428	533.93	998	681.818	1009	1751.5	1389.36
Volume (cm^3^)	308	465	386.25	499	915.60	627.954	905	1550	1240.500
Density (g/cm^3^)	1.04	1.06	1.05	1.07	1.09	1.08	1.11	1.13	1.12
A_C_ (cm^2^)	532.99	701.34	620.165	746.52	1136.20	876.29	1132.58	1631.30	,402.73
Shape coefficient	11.68	11.68	11.68	11.86	12.05	11.95	12.10	12.18	12.15

Note

Average projected area (cm^2^).

**Table 2:** Mean comparison results of data from hydrodynamic properties analysis of sugar beet samples at three mass levels

Parameters	m < 500 (g)		500 < m<1,000 (g)		m >1,000	
	Mean	CV (%)	Mean	CV (%)	Mean	CV (%)
V_t_ (m/s)	0.12 ± 0.01^a^	8.35	0.16 ± 0.02^b^	4.32	0.22 ± 0.02^c^	6.23
T_d_ (s)	14.26 ± 0.04^a^	5.36	10.92 ± 0.03^b^	5.89	7.94 ± 0.04^c^	5.42
F_b_ (*N*)	3.79 ± 0.03^c^	5.24	6.30 ± 0.02^b^	6.12	12.03 ± 0.03^a^	8.45
F_d_ (*N*)	0.20 ± 0.03^c^	7.53	0.29 ± 0.04^b^	7.14	1.49 ± 0.03^a^	6.12

Abbreviations

V_t_ = terminal velocity, T_d_ = falling time, F_b_ = buoyancy force, F_d_ = drag force, CV = the coefficient of variation.

Reported values correspond to the mean ± standard deviation. Different letters in the same row indicate significant differences (*p ≤ .05*).

### 3.2 Terminal velocity

#### 3.2.1 Evaluation of terminal velocity based on KHAT4 model

Since the value of the Reynolds’ number is N_Re_ > 1, the Equation 17 (KHAT4 model) was used to calculate the terminal velocity (Kheiralipour et al., 2008, 2009). According to this equation and according to Table 3, seven models were adjusted and the coefficients related to those models were determined. According to Table 3, in model 1, the effect of three physical factors, namely the water–sugar beet density difference, the shape coefficient, and volume with each other, in models 2, 3, and 4, the effect of each physical factor including the water–sugar beet density difference, volume, and shape coefficient of beet separately, and in models 5, 6, and 7, the effect of water–sugar beet density difference with shape coefficient, the effect of water–sugar beet density difference with volume, and volume effect with shape coefficient (as two factor) were examined at the terminal velocity. During the modeling process, the aforementioned models were fitted to the data by nonlinear regression. In addition to the R^2^ coefficient, three RMSE, χ^2^, and P‐value indicators were used to determine the suitability of the fit and determine the best model. Among the models presented, model 6, which had the highest R^2^ value and the lowest values of RMSE, χ^2^, and P‐value, was selected as the best model.

**Table 3:** Fitting experimental data into the KHAT4 model and determination of model coefficients

Row	Model	A	b	c	d	E	R^2^	RMSE	^2χ^	*p‐value* (%)
1	*F*(V_t_)=*F*($\rho_{f}‐\rho_{w}$, v, S_h_)	0.81	0.15	0.20	0.02	0.02	0.70	0.23	0.12	4.11
2	*F*(V_t_)=*F*($\rho_{f}‐\rho_{w}$)	0.31	0.28	0.00	0.00	0.01	0.74	0.21	0.08	4.05
3	*F*(V_t_)=*F*(v)	1.37	0.00	0.35	0.00	0.045	0.77	0.18	0.08	3.68
4	*F*(V_t_)=*F*(S_h_)	0.21	0.00	0.00	−0.18	0.031	0.51	0.32	0.24	6.85
5	*F*(V_t_)=*F*($\rho_{f}‐\rho_{w}$, S_h_)	1.20	0.71	0.00	−0.15	0.02	0.59	0.30	0.25	6.08
6	*F*(V_t_)=*F*($\rho_{f}‐\rho_{w}$ ,v)	1.11	0.09	0.25	0.00	0.01	0.79	0.18	0.06	2.56
7	*F*(V_t_)=*F*(v, S_h_)	1.69	0.00	0.23	−0.31	0.01	0.73	0.22	0.11	1.01

Abbreviations

RMSE, root mean square error; $X^{2}$, chi‐square test; *p*‐value, probability value.

In other words, according to this model, the volume and the water–sugar beet density difference were determined as the most important parameters affecting the sugar beet terminal velocity. It is noteworthy that the greater numerical value of the volume index than the water–sugar beet density difference indicates that the volume index has a greater effect on the terminal velocity than water–sugar beet density difference. According to the results, the shape coefficient had the least effect on the value of the terminal velocity. Several studies on this topic have calculated terminal velocities for apple (Kheiralipour et al., 2008), apricot (Mirzaee et al., 2009), tomato (Taheri Garavand et al., 2010), lime (Rozbahani et al., 2019), and cantaloupe (Jahanbakhshi et al., 2019).

#### 3.2.2 Evaluation of terminal velocity based on Jordan and Clark's model

The Jordan and Clark equation (Equation 18) was then used to model the terminal velocity of sugar beet specimens (Jordan & Clark, 2004). In this equation, the mass and the water–sugar beet density difference are considered as the parameters affecting the terminal velocity. In this regard, five models were adapted (Table 4) and the coefficients related to the models were determined. According to Table 4, in model 1 the effect of mass alone, in models 2 and 3 the effect of water–sugar beet density difference alone but either without or with, respectively, considering the constant coefficient (D) and in models 4 and 5 the simultaneous effect of mass and water–sugar beet density difference, but either without or with, respectively, considering the constant coefficient (D) on the value of the terminal velocity were examined. Then, nonlinear regression method was used to fit the data and the mentioned models were fitted with the data. In addition to the R^2^ coefficient, three RMSE, χ^2^, and P‐value indicators were used to determine the suitability of the fit and determine the best model.

**Table 4:** Fitting experimental data into the Jordan and Clark's model and determination of model coefficients

***p‐value* (%)**	χ**^2^**	**RMSE**	**R^2^**	**D**	**c**	**b**	**A**	**Model**	**Row**
2.29	0.06	0.25	0.68	0.01	0.00	0.63	0.17	*F*(V_t_)=*F*(m)	1
6.89	0.11	0.48	0.57	0.00	0.51	0.00	0.59	*F*(V_t_)=*F*($\rho_{f}‐\rho_{w}$)	2
6.52	0.10	0.39	0.54	0.02	0.32	0.00	0.29	*F*(V_t_)=*F*($\rho_{f}‐\rho_{w}$)	3
4.36	0.08	0.32	0.61	0.00	0.15	0.38	0.26	*F*(V_t_)=*F*($\rho_{f}‐\rho_{w}$, m)	4
2.35	0.04	0.15	0.76	0.01	0.08	0.30	0.21	*F*(V_t_)=*F*($\rho_{f}‐\rho_{w}$, m)	5

Abbreviations

RMSE, root mean square error; $X^{2}$, chi‐square test; *p*‐value, probability value.

According to Table 4, model 1 had the highest R^2^ value and the lowest values of RMSE, χ^2^, and P‐value compared to models 2 and 3. This result shows that sugar beet mass has a greater effect on the terminal velocity than the water–sugar beet density difference. On the other hand, according to the results obtained and among the fitted models, model 5 had the highest R^2^ value and the lowest RMSE, χ2, and P‐value amounts. Of course, the greater numerical value of the mass index greater than the water–sugar beet density difference indicates that this index has a greater effect on the terminal velocity than the difference between water–sugar beet density.

As mentioned, according to Tables 3 and 4, volume and mass had a greater effect on the terminal velocity than the water–sugar beet density difference, and models 5 and 6 were selected from Table 4 and 3, respectively, as the best model. Since mass and volume can be converted to each other, it can be concluded that these two models (5 and 6) obtained from the KHAT4 and Jordan and Clark equations, respectively, are equal. According to studies, many researchers have achieved similar results by researching different agricultural products (Hasanpour Kahnamuyi & Ghaffari, 2013; Kheiralipour et al., 2009; Naderi‐Boldaji et al., 2008; Rozbahani et al., 2019; Tab atabaeefar & Rajabipour, 2005; Taheri Garavand et al., 2010).

### 3.3 Evaluation of falling time

#### 3.3.1 Evaluation of falling time based on KHAT4 model

Equation 21 was used to model the falling time of sugar beet samples. According to this equation and according to Table 5, seven models were used to predict the falling time of samples. The parameter E was introduced because the samples did not reach their terminal velocity in a short time.

**Table 5:** Fitting experimental data into the KHAT4 model and determination of model coefficients

**Row**	**Model**	**A**	**b**	**c**	**d**	**E**	**R^2^**	**RMSE**	χ**^2^**	***p‐value* (%)**
1	*F*(T_d_)=*F*(v)	8.29	0.00	0.85	0.00	1.50	0.63	0.053	0.08	3.31
2	*F*(T_d_)=*F*(S_h_)	17.33	0.00	0.00	0.23	1.14	0.48	0.261	0.26	7.42
3	*F*(T_d_)=*F*($\rho_{f}‐\rho_{w}$)	2.16	0.62	0.00	0.00	0.20	0.62	0.065	0.07	3.28
4	*F*(T_d_)=*F*($\rho_{f}‐\rho_{w}$ ,v)	3.56	0.39	0.52	0.00	0.35	0.71	0.042	0.03	1.22
5	*F*(T_d_)=*F*($\rho_{f}‐\rho_{\text{fw}}$, S_h_)	5.66	0.28	0.00	0.09	1.63	0.58	0.161	0.11	6.07
6	*F*(T_d_)=*F*(v, S_h_)	8.66	0.00	0.58	0.06	2.52	0.57	0.157	0.18	6.09
7	*F*(T_d_)=*F*($\rho_{f}‐\rho_{w}$, v, S_h_)	5.89	0.15	0.33	0.03	2.50	0.63	0.088	0.04	2.89

Abbreviations

RMSE, root mean square error; $X^{2}$, chi‐square test; *p*‐value, probability value.

According to Table 5, in models 1, 2, and 3 separately, the effect of physical factors including volume, shape coefficient, and difference of water–sugar beet density and in models 4, 5, and 6 the effect of water–sugar beet density difference along with volume, the effect of water–sugar beet density difference along with shape coefficient and volume effect with shape coefficient and in model 7, the effect of three physical factors, namely water–sugar beet density difference, shape coefficient, and volume together were examined on falling time of samples. Also, nonlinear regression method was used to fit the data and the mentioned models were fitted with the data. According to the fittings, model 1 was the best among models 1, 2, and 3; on the other hand, model 4 was the best among models 3, 4, and 5. The results showed that the volume and difference of the water–sugar beet density have a greater impact on falling time than shape coefficient. In general, considering the R^2^, RMSE, χ2, and P‐value indicators, model 4 was selected as the best fit and model. Mirzaee et al. (2009) considered hydro‐sorting of apricot fruits based on some of hydrodynamic properties. According to the results, the difference of water‐apricot density had a greater effect on the falling time of the samples than the volume of fruits. Taheri Garavand et al. (2010) studied using the rising time of tomato through water as a means of hydro‐sorting. According to the results, the difference of water‐tomato density had major effect on rising time.

#### 3.3.2 Evaluation of falling time based on Jordan and Clark's model

Equation 22 was used to model the falling time of sugar beet specimens based on the Jordan and Clark equation. According to this equation and according to Table 6, five models were adjusted and the coefficients related to those models were determined. According to this theory, the parameters affecting the falling time of a product are stated as the mass and the water–sugar beet density difference; but in this model, the effect of the shape coefficient is not considered. In the following, based on the equation of Jordan and Clark, five equations according to Table 6 were used to model the falling time of sugar beet samples. As shown, parameters A and D were excluded from some of the models.

**Table 6:** Fitting experimental data into the Jordan and Clark's model and determination of model coefficients

***P‐ value* (%)**	**^2^χ**	**RMSE**	**R^2^**	**D**	**c**	**b**	**A**	**Model**	**Row**
3.52	0.25	0.06	0.63	1.20	0.00	−0.18	9.72	*F*(V_t_)=*F*(m)	1
3.54	0.24	0.08	0.61	0.83	−0.55	0.00	2.50	*F*(V_t_)=*F*($\rho_{f}‐\rho_{w}$)	2
6.33	0.32	0.07	0.59	0.00	−0.55	0.00	2.88	*F*(V_t_)=*F*($\rho_{f}‐\rho_{w}$)	3
3.54	0.21	0.05	0.67	0.38	−0.47	−0.73	2.90	*F*(V_t_)=*F*($\rho_{f}‐\rho_{w}$, m)	4
3.47	0.24	0.05	0.65	0.00	−0.11	−0.58	7.44	*F*(V_t_)=*F*($\rho_{f}‐\rho_{w}$, m)	5

Abbreviations

RMSE, root mean square error; $X^{2}$, chi‐square test; *P*‐value, probability value.

According to Table 6, and considering the evaluation criteria of the models, the mass of the tubers had a greater effect on the falling time of the samples than the difference of water–sugar beet density. According to Table 7, the greater effect of mass than the water–sugar beet density difference on the falling time of samples can be related to greater changes of mass coefficient of variation (0.65) than the coefficient of variation of water–sugar beet density difference (0.44). Also, according to Table 6, model 4 had the best fit compared to other models due to having the highest R^2^, and the lowest values of RMSE, χ2, and P‐value, and was selected as the best model (Hasanpour Kahnamuyi & Ghaffari, 2013; Kheiralipour et al., 2009; Rozbahani et al., 2019; Tab atabaeefar & Rajabipour, 2005; Taheri Garavand et al., 2010).

**Table 7:** Coefficient of variations of sugar beet properties

**Variable**	**Min**	**Mean**	**Max**	**CV (%)**
($\rho_{f}‐\rho_{w}$) g/cm^3^	0.04	0.085	0.13	0.44
$\left(v\right)$ cm^3^	308.08	929.20	1,550.32	0.56
$\left(m\right)$ g	322.41	1,036.76	1751.12	0.65
$S_{h}$	11.68	11.93	12.18	0.12

Abbreviations

CV, the coefficient of variation; *SD*, standard deviation.

## 4 CONCLUSIONS

In this research, the terminal velocity and falling time of sugar beet were theoretically formulated and then measured experimentally using water column. According to the equations of KHAT4 and Jordan and Clark, the parameters of mass, volume, and the difference of water–sugar beet density had the highest but the shape coefficient had the least effect on the terminal velocity and falling time. Therefore, it seems that different samples of sugar beet can be separated based on their density.

## CONFLICT OF INTEREST

The authors declare that they do not have any conflict of interest.

## ETHICAL APPROVAL

This study does not involve any human or animal testing.
